# The role of environmental stress and DNA methylation in the longitudinal course of bipolar disorder

**DOI:** 10.1186/s40345-019-0176-6

**Published:** 2020-02-12

**Authors:** Ashley L. Comes, Darina Czamara, Kristina Adorjan, Heike Anderson-Schmidt, Till F. M. Andlauer, Monika Budde, Katrin Gade, Maria Hake, Janos L. Kalman, Sergi Papiol, Daniela Reich-Erkelenz, Farah Klöhn-Saghatolislam, Sabrina K. Schaupp, Eva C. Schulte, Fanny Senner, Georg Juckel, Max Schmauß, Jörg Zimmermann, Jens Reimer, Eva Reininghaus, Ion-George Anghelescu, Carsten Konrad, Andreas Thiel, Christian Figge, Martin von Hagen, Manfred Koller, Detlef E. Dietrich, Sebastian Stierl, Harald Scherk, Stephanie H. Witt, Sugirthan Sivalingam, Franziska Degenhardt, Andreas J. Forstner, Marcella Rietschel, Markus M. Nöthen, Jens Wiltfang, Peter Falkai, Thomas G. Schulze, Urs Heilbronner

**Affiliations:** 1 Institute of Psychiatric Phenomics and Genomics, University Hospital, LMU Munich, Nussbaumstrasse 7, 80336 Munich, Germany; 2 International Max Planck Research School for Translational Psychiatry (IMPRS-TP), 80804 Munich, Germany; 3 Department of Translational Research in Psychiatry, Max Planck Institute of Psychiatry, 80804 Munich, Germany; 4 Department of Psychiatry and Psychotherapy, University Hospital, LMU Munich, 80336 Munich, Germany; 5 Department of Psychiatry and Psychotherapy, University Medical Center Göttingen, 37075 Göttingen, Germany; 6 Department of Neurology, Klinikum rechts der Isar, School of Medicine, Technical University of Munich, 81675 Munich, Germany; 7 Department of Psychiatry, Ruhr University Bochum, LWL University Hospital, 44791 Bochum, Germany; 8 Department of Psychiatry and Psychotherapy, Bezirkskrankenhaus Augsburg, University of Augsburg, 86156 Augsburg, Germany; 9 Psychiatrieverbund Oldenburger Land gGmbH, Karl-Jaspers-Klinik, 26160 Bad Zwischenahn, Germany; 10 Department of Psychiatry and Psychotherapy, University Medical Center Hamburg-Eppendorf, 20246 Hamburg, Germany; 11 Department of Psychiatry and Psychotherapeutic Medicine, Research Unit for Bipolar Affective Disorder, Medical University of Graz, 8036 Graz, Austria; 12 Department of Psychiatry, Dr. Frontheim-Mental Health, 38704 Liebenburg, Germany; 13 Department of Psychiatry and Psychotherapy, Agaplesion Diakonieklinikum, 27356 Rotenburg, Germany; 14 Karl-Jaspers Clinic, European Medical School Oldenburg-Groningen, 26160 Oldenburg, Germany; 15 Clinic for Psychiatry and Psychotherapy, Clinical Center Werra-Meißner, 37269 Eschwege, Germany; 16 Asklepios Specialized Hospital, 37081 Göttingen, Germany; 17 AMEOS Clinical Center Hildesheim, 31135 Hildesheim, Germany; 18 Center für Systems Neuroscience (ZSN) Hannover, 30559 Hannover, Germany; 19 Department of Psychiatry, Medical School of Hannover, 30625 Hannover, Germany; 20 Psychiatric Hospital Lüneburg, 21339 Lüneburg, Germany; 21 AMEOS Clinical Center Osnabrück, 49088 Osnabrück, Germany; 22 Department of Genetic Epidemiology in Psychiatry, Central Institute of Mental Health, Medical Faculty Mannheim, University of Heidelberg, 68159 Mannheim, Germany; 23 Institute of Human Genetics, University of Bonn, School of Medicine & University Hospital Bonn, 53127 Bonn, Germany; 24 Center for Human Genetics, University of Marburg, 35033 Marburg, Germany; 25 Department of Biomedicine, University of Basel, 4031 Basel, Switzerland; 26 Department of Psychiatry (UPK), University of Basel, 4002 Basel, Switzerland; 27 German Center for Neurodegenerative Diseases (DZNE), 37075 Göttingen, Germany; 28 iBiMED, Medical Sciences Department, University of Aveiro, 3810-193 Aveiro, Portugal

**Keywords:** DNA methylation, Bipolar disorder, Stressful life events, Longitudinal, Epigenomics, Epigenetic aging

## Abstract

**Background:**

Stressful life events influence the course of affective disorders, however, the mechanisms by which they bring about phenotypic change are not entirely known.

**Methods:**

We explored the role of DNA methylation in response to recent stressful life events in a cohort of bipolar patients from the longitudinal PsyCourse study (*n* = 96). Peripheral blood DNA methylomes were profiled at two time points for over 850,000 methylation sites. The association between impact ratings of stressful life events and DNA methylation was assessed, first by interrogating methylation sites in the vicinity of candidate genes previously implicated in the stress response and, second, by conducting an exploratory epigenome-wide association analysis. Third, the association between epigenetic aging and change in stress and symptom measures over time was investigated.

**Results:**

Investigation of methylation signatures over time revealed just over half of the CpG sites tested had an absolute difference in methylation of at least 1% over a 1-year period. Although not a single CpG site withstood correction for multiple testing, methylation at one site (cg15212455) was suggestively associated with stressful life events (*p* < 1.0 × 10^−5^). Epigenetic aging over a 1-year period was not associated with changes in stress or symptom measures.

**Conclusions:**

To the best of our knowledge, our study is the first to investigate epigenome-wide methylation across time in bipolar patients and in relation to recent, non-traumatic stressful life events. Limited and inconclusive evidence warrants future longitudinal investigations in larger samples of well-characterized bipolar patients to give a complete picture regarding the role of DNA methylation in the course of bipolar disorder.

## Background

Bipolar disorder (BD) remains an interesting candidate for neurobiological analyses owing to its heterogenous presentation and both genetic and environmental risk factors (Ludwig and Dwivedi 2016). While genome-wide association studies (GWAS) in BD have identified dozens of associated variants, they have explained only a small fraction of overall disease liability (Stahl et al. 2019). Therefore, the last decade has seen a shift towards investigating the complex interplay between genetic and environmental risk factors (Sharma et al. 2016). Advances in technologies have supported high-throughput investigations of biological markers representative of environmental modulation of the genome. These biomarkers hold promise for stratifying symptom-based phenotypes and assessing the prognosis of individual patients (Kobeissy et al. 2012). Moreover, these biomarkers could contribute to a more accurate multi-level diagnostic framework which relies on biological measures to supplement clinical ratings of symptoms (Meana and Mollinedo-Gajate 2017).

BD is a chronic, disabling, and severe mental illness characterized by recurrent depressive and manic episodes, somatic and psychiatric comorbidities, and functional impairments (Goodwin and Jamison 2007). Considering the high global burden and lifetime prevalence of bipolar spectrum disorders, estimated at approximately 2.4% (Rowland and Marwaha 2018), there is a need to better understand the factors affecting its onset and course. The significance of environment, especially childhood trauma and stressful life events on the trajectories of affective disorders, including vulnerability, onset, relapse and occurrence, has been well established (Aldinger and Schulze 2017; Lex et al. 2017; Johnson 2005; Alloy et al. 2005; Paykel 2003). However, little is known about the mechanisms involved in the consequences of such life events.

Recently, emphasis has been placed on the potential role of epigenetic variation in the etiopathogenesis of BD (Li et al. 2015). Epigenetics is an adaptive mechanism which can modulate the stress response through subtle gene expression modifications (Aas et al. 2016). In particular, DNA methylation (DNAm), the addition of a methyl group to DNA, primarily at cytosine-guanine dinucleotides (CpG), may pose a “mechanism by which life-experiences become ‘embedded’ in the genome” (Marzi et al. 2018).

Increasing evidence from both animal and human data supports the epigenetic programming of genes in response to trauma and chronic stress. Consistent findings have linked prenatal (Monk et al. 2012; Weaver et al. 2004) and early-life adversities to epigenetic modifications of genes, especially those involved in the hypothalamic–pituitary–adrenal (HPA) axis (Kular and Kular 2018; McGowan et al. 2009; Vinkers et al. 2015; Jaworska-Andryszewska and Rybakowski 2019). While several studies have shown methylation changes associated with trauma during the adult period, few studies have investigated non-traumatic chronic stress (Matosin et al. 2017) or acute stressful life events. Candidate gene approaches in the general population have reported differential methylation of CpGs in the vicinity of *SLC6A4* (Alasaari et al. 2012), *TH* (Myaki et al. 2015), and *BDNF* (Song et al. 2014) in association with sustained work-related stress. One study, which examined *LINE*-*1* as a proxy for global methylation, found no signification associations with chronic lifestyle stress (Duman and Canli 2015). To the best of our knowledge, not a single study has explored epigenome-wide signatures of DNAm in relation to acute, non-traumatic stress in humans. With regards to BD, studies have investigated methylation differences as both trait and state markers of the disorder in several promoter regions including *SLC6A4*, *PPIEL31*, *BDNF*, *HCG9*, *KCNQ3*, *5HTR1A* and *GPR24* (Ludwig and Dwivedi 2016; Fries et al. 2016; Pishva et al. 2014). Interestingly, evidence supports altered DNAm profiles for high-risk affected and even unaffected offspring of individuals with BD in comparison to low risk controls. Moreover, there seems to be a unique rate of change in DNAm over time for high risk individuals (Duffy et al. 2019). However, despite findings of differential epigenetic profiles, results have been inconsistent and there remains a need for genome-wide methylation studies, especially ones longitudinal in design.

This study aims to gain a better understanding of the role of epigenetic modifications, specifically DNAm, in relation to stress during the course of BD. Using repeated measures over a 1-year period, we explored the relationship between DNAm and stressful life events in chronic BD patients. We took a two-pronged approach, first by interrogating CpGs in the vicinity of candidate genes previously implicated in the stress response and, second, by conducting an exploratory epigenome-wide analysis. Furthermore, we determined whether changes in symptom and stress measures over time were associated with a DNAm-based age estimate and epigenetic aging.

## Methods

### Study sample

The study was conducted using data from the longitudinal PsyCourse cohort. PsyCourse has been described in detail (Budde et al. 2019). Briefly, PsyCourse is a multi-site, naturalistic study, based in the German and Austrian population. Psychopathology, pharmacological treatment, childhood trauma and current stressful life events were among other variables assessed at each of four visits (6-month intervals). Likewise, peripheral blood samples were collected at each visit, paving the way for a detailed analysis of the longitudinal correlation between disease status and peripheral biomarkers. For the purpose of this study, a subset of PsyCourse participants (*n* = 96) was selected according to a DSM-IV diagnosis (American Psychiatric Association 2002) of type I or II BD, availability of genotype data and biomaterial, and completed childhood trauma and stressful life events questionnaires. Demographic and clinical characteristics of these patients are reported in Table 1. The study was approved by the local ethics committee for each study center and was carried out following the rules of the Declaration of Helsinki. All individuals provided written informed consent.

**Table 1:** Sample demographic and clinical characteristics

	Baseline (*n* = 96)	1-year follow-up (*n* = 95)	*p*-value
Sex			
Female	50	50	
Age, mean ± SD	45.2 ± 12.4	46.17 ± 12.4	
Duration of illness, mean ± SD	13.52 ± 11.8	14.66 ± 11.8	
DSM-IV diagnosis			
BD-I	79	78	
BD-II	17	17	
Medication			
Combo therapy	81	75	
Monotherapy	14	16	
No meds	1	4	
Childhood trauma (yes)	48	48	
LEQ scores, mean ± SD			
Bad events	10.2 ± 13.8	6.3 ± 6.6	0.004^b^
Good events	9.7 ± 10.2	8.4 ± 7.6	0.191^b^
Total events	19.9 ± 18.4	14.1 ± 10.7	0.001^b^
Symptom ratings			
GAF, mean ± SD	61.5 ± 12.6	65.8 ± 12.4	0.032^a^
YMRS sum, mean ± SD	3.9 ± 5.8	2.4 ± 3.7	0.216^b^
IDS-C_30_, mean ± SD	13.7 ± 11.0	10.6 ± 9.7	0.124^b^
PANSS sum, mean ± SD	42.8 ± 11.8	39.2 ± 9.6	0.063^b^

^a^Paired sample t-test

^b^Wilcoxon signed rank test

### Measures

#### Stressful life events

Current stressful life events were assessed with the Life Events Questionnaire (LEQ), a 79-item self-report instrument that has been described in detail (Norbeck 1984; Sarason et al. 1978). The LEQ covers a wide range of stressor exposure related to health, work, school, residence, love and marriage, family and friends, parenting, the personal sphere or social environment, finances, crime and legal matters. At each visit, participants reported whether they experienced any of the listed events in the last 6 months. When the patient experienced a specific event, they rated: (1) the nature of the event (good/bad) and (2) the impact of the event on his/her life (0–3). At each time point, adverse life events were summed to yield a stress score that reflects the impact ratings of all “bad” events. The same was done for the impact ratings of “good” events. A total score was also summed including impact ratings of both “bad” and “good” events. These three LEQ scores were used as outcome measures in our association analyses.

#### Childhood trauma

The Childhood Trauma Screener (CTS) is a German, short version of the Childhood Trauma Questionnaire (Bernstein et al. 1997, 2003; Grabe et al. 2012). The screener includes five questions to assess sexual, physical and emotional abuse, as well as emotional and physical neglect. Validated threshold values (Glaesmer et al. 2013) were used to transform ratings for each item into a dichotomous scale in order to identify individuals with reported childhood trauma (yes/no). Details on reported childhood trauma and thresholds used can be found in Additional file 1: Table S1.

#### Symptom ratings

The Positive and Negative Syndrome Scale (PANSS) was used as a measure of psychopathology at the time of testing (Kay et al. 1987). A continuous total score of the three subscales, i.e. positive, negative, and general symptoms was used. The Global Assessment of Functioning (GAF) score was used as a measure of psychosocial functioning (Luborsky 1962; Endicott et al. 1976). The Young Mania Rating Scale (YMRS) was used as a measure of manic symptoms in the last 48 h (Young et al. 1978). Lastly, the Inventory of Depressive Symptomatology (IDS-C_30_), a 30-item rating scale, was used to assess the severity of depressive symptoms (Trivedi et al. 2004).

### Analysis of DNA methylation

#### DNA samples

Genomic DNA was extracted from whole blood using the PerkinElmer Chemagen Kit (chemagic DNA Blood10k prefilling VD120419.che) and all samples were subsequently stored in a Hamilton Bios M system at − 80 °C. DNA quality was assessed using the QIAxcl^®^ system. DNA samples from baseline and 1-year follow-up visits were used to obtain methylation data. Prior to downstream analyses, potential population stratification was evaluated, and an initial step to remove European population outliers was taken (Budde et al. 2019). Thus, our sample consists of an ethnically homogenous population of Caucasians of European descent.

#### Illumina EPIC chip processing

Bisulfide conversion of DNA and processing of methylation arrays was accomplished in collaboration with the Institute of Human Genetics, University of Bonn, Germany. Whole-blood genomic DNA diluted with water (50 ng/μl) was treated with sodium bisulfite using the EpiTect^®^ Bisulfite Kit from QIAGEN^®^ following the manufacturer’s protocol. DNAm was assessed using the Illumina Infinium Human MethylationEPIC BeadChip array (Illumina Inc., San Diego, CA, USA) according to the manufacturer’s instructions. To minimize batch effects during DNAm measurement, an algorithm for sample randomization was used for positioning samples onto 96-well plates according to exposures of interest and confounding variables (see Additional file 1).

### Quality control and normalization

#### Quality control

The Bioconductor R package *minfi* was used to read raw intensity data files (.idat files) into R and for the subsequent quality control and normalization of methylation data (Aryee et al. 2014). Concordance between methylation-predicted and reported sex was confirmed. Filtering of poor-performing samples and probes was performed (see Additional file 1: Table S2). Probes with low detection *p*-values (> 0.05 in > 10% of samples) were excluded. Using the function dropLociWithSnps(), SNPs inside the probe body and at the nucleotide extension were removed according to a minor allele frequency ≥ 5% based on dbSNP. To prevent a possible gender effect, X and Y chromosomes were removed. According to a list previously published (Chen et al. 2013), non-specific probes i.e. probes on the EPIC array that co-hybridize to alternate genomic sequences, were removed. Lastly, probes with a bead count < 3 were removed.

#### Normalization

Data were normalized using functional normalization (FunNorm), an extension of quantile normalization. FunNorm uses internal control probes present on the array to infer between-array technical variation, by default using the first two principal components of the control probes (Fortin et al. 2014). Density plots were used to evaluate the distribution of *M*-values before and after functional normalization (see Additional file 1: Fig. S1).

Technical batch effects were then identified using linear regressions to inspect the association of principal components of the methylation values with possible technical batches. Additionally, the R package *shinyMethyl* was used for visual inspection of principle component analysis (PCA) plots. Identified batch effects (i.e., array and slide) were removed using the Empirical Bayes’ method *ComBat* (Johnson et al. 2007). Batch corrected *M*-values after *ComBat* were used for downstream analyses (see Additional file 1: Fig. S2). According to inspection of PCA plots, a single sample remained an outlier after batch correction and was excluded.

#### Confounders

Considering cell-type composition is a confounding factor in epigenome-wide association studies (EWAS), the *minfi* function estimateCellcounts() was used to estimate the cell type composition for our samples. This function uses a modified version of the Houseman algorithm to obtain a cell counts vector for the six cell-types (i.e., CD4T, CD8T, NK, B cells, monocytes, and granulocytes) for each sample (Houseman et al. 2012).

Active smoking is another established modifier of DNA methylation (Lee and Pausova 2013). Methylation-based smoking scores were calculated based on the methylation profile of the 187 CpG sites identified in Zeilinger et al. (2013). First, raw beta values were normalized using the Teschendorff et al. beta-mixture quantile dilation (BMIQ) strategy (Teschendorff et al. 2013). Adjusted beta-values were then used for calculation of methylation-based smoking scores using methods previously described (Elliott et al. 2014). The correlation between self-reported number of cigarettes smoked yearly and methylation-based smoking scores was assessed (Spearman’s ρ = 0.64; *p* < 0.001).

To rule out possible confounding effects of medication, 5 samples were excluded in sensitivity analyses. These samples were participants who were not taking psychotropic drugs at the time of testing. All other participants were taking at least one (monotherapy) or a combination (combo therapy) of the following (1) antidepressants, (2) antipsychotics, (3) mood stabilizers, (4) tranquilizers, or (5) other psychiatric medications.

### Statistical analyses

All statistical analyses were performed in R version 3.4.4 (http://www.r-project.org/) (R Core Team 2014).

#### Change in methylation over time

The general “stability” of methylation over time was investigated. First, the absolute change in methylation *β*-values between baseline and 1-year follow-up visits were calculated across all CpG sites. To determine whether differential methylation between visits remained significant after adjusting for known confounders, the package *lme4* (Bates et al. 2015) was used to fit a linear mixed-effects model (LMM) with the dependent variable “*M*-value” and the independent variable “time”, adjusting for age, sex, DNAm smoking scores, and cell composition estimates. Patient ID was included as the random effect term.

#### Candidate gene analysis

The association between LEQ scores and the interaction between CT and total LEQ scores with DNAm was assessed via LMMs, adjusting for covariates as described above. We interrogated DNAm in the vicinity of genes previously implicated in the HPA-axis (i.e. *BDNF*, *FKBP5*, *IL6*, *SLC6A4*, and *OXTR*). All probes on the EPIC array annotated to each of these five genes were identified. The number of probes per gene ranged from 22 to 124. We corrected for multiple testing on a gene-level by applying the false discovery (FDR) correction (Benjamini and Hochberg 1995) per gene, with FDR-corrected *p*-values ≤ 0.05 deemed significant. Afterwards, Bonferroni-correction was used to correct overall for the number of candidate-genes tested.

#### Exploratory EWAS

An exploratory EWAS was conducted. As a means of noise reduction, the top 10% of the most variable CpGs of the normalized, batch corrected *M*-values were extracted according to median absolute deviation (MAD) scores i.e. the median of the absolute deviations from the data’s median. Associations between the most variable sites and LEQ scores and the interaction between childhood trauma and total LEQ scores were then tested using LMMs, adjusting for covariates as described above.

#### Epigenetic aging

DNAm-based age prediction was performed using the Horvath age estimation algorithm (Horvath 2013) with a freely available online tool (https://dnamage.genetics.ucla.edu/home) which predicts DNAm-age based on the methylation of 353 CpGs using an elastic net penalized regression model. The difference between the estimated epigenetic age and chronological age (Δage) and a measure of epigenetic age acceleration (AA), i.e., the residual from regressing DNAm age on chronological age, were calculated. LMMs were used to determine the effect of LEQ scores on Δage, adjusting for chronological age, sex, DNAm smoking scores, cell composition estimates, and technical batch effects (sample slide and array). Additionally, the difference in symptom ratings and stress scores between visits were calculated. The association between the change in symptoms and LEQ scores between baseline and 1-year follow-up with AA at 1-year follow-up was determined via linear regression models, again controlling for chronological age, sex, DNAm smoking scores, cell composition estimates and technical batch effects.

#### Additional analyses

Nominally significant CpGs (unadjusted *p* < 0.05) associated with total LEQ scores were used for gene-based enrichment analysis using the *GOmeth* function from the Bioconductor package *missMethyl*. *GOmeth* maps a vector of CpG sites to Entrez Gene IDs, and tests for gene ontology (GO) term pathway enrichment using a hypergeometric test (Geeleher et al. 2013). Additionally, the correlation between DNAm in blood and four brain regions was explored for the most suggestive CpGs associated with total LEQ scores (see Additional file 1).

## Results

### Change in methylation over time

**Fig. 1:**
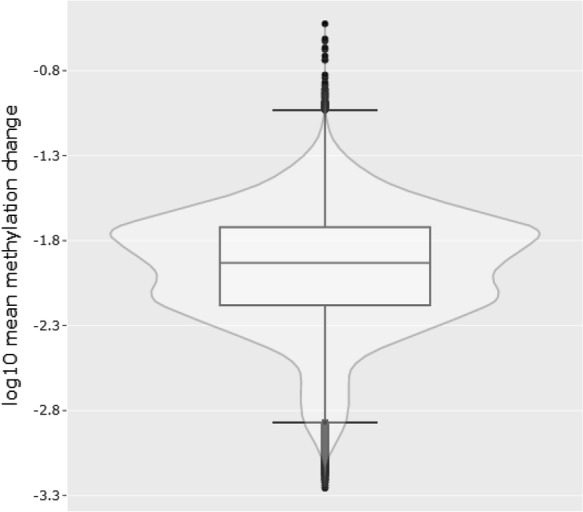
Boxplot depicting the log10 mean change in methylation (β) between baseline and 1-year follow-up visit

**Fig. 2:**
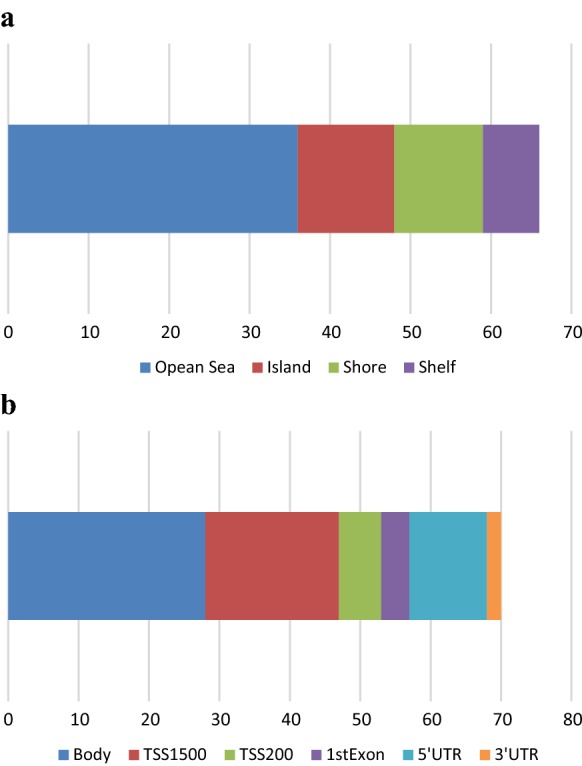
Functional genomic distribution for the least stable CpG sites over 1-year period (|Δβ| ≥ 0.10). **a** Depicts the distribution of probes that fell within CpG Islands (12/66), Shelves (7/66), Shores (11/66) and the Open Sea (36/66). **b** Depicts the distribution of probes that fell within the gene body (28), 5’ UTR (11), 3’ UTR (2), 1st Exon (4), TSS 1500 (19) and TSS 200 (6)

The mean absolute difference in methylation (β) between visits 1 and 3 (|Δβ|) was calculated across all samples for all CpG sites (Fig. 1). Over the 1-year period, |Δβ| ranged from < 0.001 to 0.299 with an average change of 0.014. Of 753,251 CpG sites, only 68 had an |Δβ| of 0.10 or more, while 8454 sites differed by at least 0.05 between visits. Just over half of the sites (428,610) showed an absolute difference in methylation of at least 1%. Investigation of the functional genomic distribution of the least stable CpGs over time (|Δβ| ≥ 0.10) revealed the majority of CpGs fell within Open Seas, while 12 fell within CpG Islands, and the remaining in CpG Shores and Shelves (Fig. 2). In summary, 34,776 CpG sites showed a nominally significant difference over time (unadjusted *p*-value < 0.05), after correcting for age, sex, smoking and cell composition estimates. However, not a single locus withstood correction for multiple testing (FDR-corrected *p*-value < 0.05).

### Methylation association analysis

**Fig. 3:**
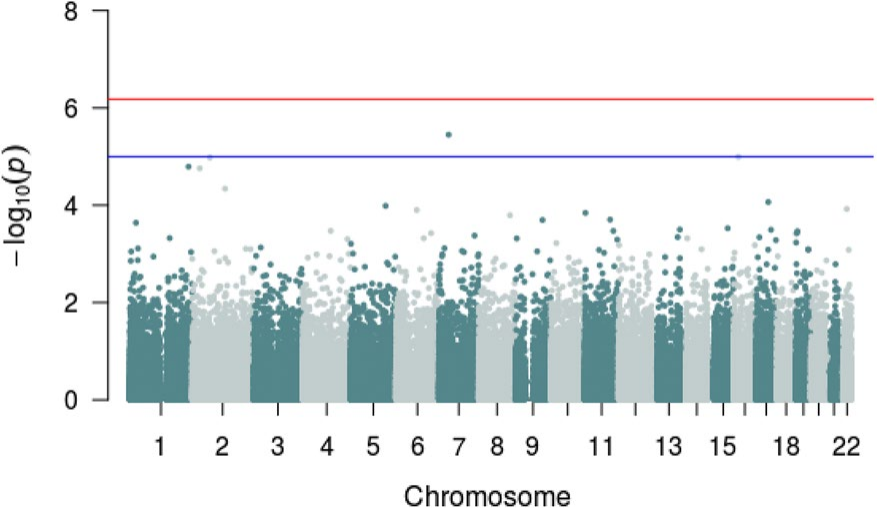
Manhattan plot for association between DNA methylation and total LEQ scores. The horizontal red line represents the epigenome-wide significant threshold for this study (*p* < 6.6 × 10^−7^) and the blue line represents a suggestive significance threshold (*p* < 1.0 × 10^−5^)

**Fig. 4:**
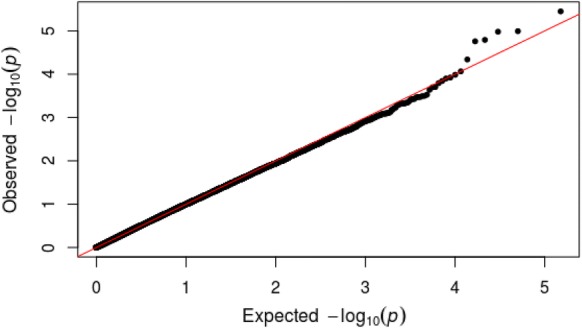
QQ plot. The plot shows no evidence for inflation or bias in the association analysis of DNA methylation with total LEQ scores (Lambda = 1.04)

We performed an exploratory analysis looking for associations between LEQ scores and DNAm in individual CpG probes in the vicinity of candidate genes previously implicated in the stress response and in the most variable CpG sites across the epigenome. Methylation at a single CpG site (cg15212455; *POU6F2*; “POU class 6 homeobox 2”; chr 7) was associated with impact ratings of total LEQ scores with a suggestive significance of *p* < 1.0 × 10^−5^, although not a single locus withstood correction for multiple testing (FDR-corrected *p* > 0.05 for all comparisons). Figure 3 shows the Manhattan plot depicting all analyzed CpG sites with their calculated *p*-values for the association between DNAm and total LEQ scores. Table 2 lists the top 20 loci associated at nominal significance with total LEQ scores. Inspection of quantile–quantile (QQ) plots did not show evidence for inflation or bias (Fig. 4; Lambda factor = 0.98). Manhattan plots and associated QQ plots for additional association analyses can be found in Additional file 1: Fig. S3–S8. The sensitivity analysis, excluding subjects who did not take psychotropic drugs at the time of testing, did not yield signification associations. These results, specific to modeling the association between DNAm and total LEQ scores, are presented in Additional file 1: Figs. S9 and S10.

**Table 2:** Top 20 CpG sites associated with total LEQ scores

Probe	t value	*p*-value	FDR-corrected *p*-value	Chr	Relation to island	Annotated gene
cg15212455	− 4.87	3.56E−06	0.263	chr7	Open Sea	*POU6F2*
cg05335886	− 4.55	1.02E−05	0.263	chr16	Island	*TMC5*
cg09725915	4.54	1.05E−05	0.263	chr2	Island	
cg24511004	4.43	1.62E−05	0.263	chr1	Open Sea	
cg18110277	− 4.50	1.74E−05	0.263	chr2	Open Sea	
cg21516302	− 4.24	4.58E−05	0.575	chr2	Open Sea	
cg05180443	− 4.04	8.61E−05	0.927	chr17	Island	*CHAD; ACSF2*
cg01440452	− 3.97	1.03E−04	0.946	chr5	N Shore	*PURA*
cg26730347	− 4.00	1.20E−04	0.946	chr22	N Shore	*SLC5A1*
cg15869582	3.94	1.26E−04	0.946	chr6	S Shore	*IBTK*
cg05919744	3.95	1.44E−04	0.977	chr11	S Shore	*SLC22A18AS;SLC22A18*
cg26822318	3.86	1.61E−04	0.977	chr8	Open Sea	*FER1L6*
cg27296293	− 3.80	1.98E−04	0.977	chr11	Island	*RP11*-*748H22.1; TRPC6*
cg06334363	3.82	2.01E−04	0.977	chr9	S Shore	*RP11*-*235C23.5; FKTN*
cg00356897	− 3.79	2.30E−04	0.977	chr1	Open Sea	*RP4*-*594I10.2*
cg24795825	3.72	2.98E−04	0.977	chr15	N Shore	*MORF4L1*
cg17984201	3.69	3.17E−04	0.977	chr13	Open Sea	
cg18002447	− 3.67	3.20E−04	0.977	chr17	Island	
cg07349208	− 3.66	3.36E−04	0.977	chr4	Island	*RP11*-*380D23.2*
cg05705044	3.65	3.38E−04	0.977	chr11	S Shore	*RBM7*

### Epigenetic aging

As expected, there was a strong positive correlation between individuals’ DNAm age and chronological age (*r* = 0.941, *p* < 0.001; see Additional file 1: Fig. S11). According to Horvath’s estimate, the mean (SD, range) AA was − 0.23 years (3.71, range − 9.94 to 9.86 years) at baseline and 0.25 years (3.95, range − 8.12 to 9.43 years) at the 1-year follow-up. Between visits, the mean (SD, range) change in AA was 0.50 years (4.97, range − 10.72 to 13.85 years). Overall, no statistically significant associations between epigenetic aging and symptom or stress measures were detected.

### Additional analyses

We included genes mapped by the top CpG sites (unadjusted *p* < 0.05) associated with total LEQ scores in an enrichment analysis. No biological processes survived FDR correction (see Additional file 1: Table S3). Blood brain correlation coefficients for methylation of the top 20 loci associated with total LEQ scores (overlapping with the 450 K Beadchip array) are presented in Table 3. Eight of the top 20 most differentially methylated loci associated with total LEQ scores showed a significant correlation between methylation in the blood and methylation in at least one brain region. Methylation of the CpG site that was most strongly associated with total LEQ scores was significantly correlated with methylation in all four brain regions (*p* < 0.001; see Additional file 1: Fig. S12).

**Table 3:** Blood-brain methylation correlation for top differentially methylated CpGs associated with total LEQ scores

Probe	Blood-PFC	*p*-value	Blood-EC	*p*-value	Blood-STG	*p*-value	Blood-CER	*p*-value
cg15212455	*0.721*	*4.16E−13*	*0.731*	*4.64E−13*	*0.747*	*1.48E−14*	*0.631*	*3.71E−09*
cg05335886	− 0.086	0.467	− 0.101	0.404	− 0.145	0.213	− 0.155	0.196
cg09725915	*0.576*	*7.86E−08*	*0.532*	*1.76E−06*	*0.626*	*1.96E−09*	*0.489*	*1.49E−05*
cg21516302	*0.373*	*0.001*	*0.522*	*3.01E−06*	*0.507*	*3.46E−06*	*0.307*	*0.009*
cg05180443	0.204	0.081	*0.336*	*0.004*	*0.298*	*0.009*	0.175	0.144
cg01440452	− 0.097	0.413	0.062	0.606	− 0.119	0.309	0.020	0.870
cg26730347	*0.499*	*6.05E−06*	*0.568*	*2.33E−07*	*0.562*	*1.51E−07*	*0.493*	*1.27E−05*
cg27296293	0.131	0.265	− 0.214	0.073	0.037	0.755	*0.236*	*0.048*
cg24795825	0.016	0.894	*0.299*	*0.011*	*0.247*	*0.033*	0.121	0.317
cg18002447	0.038	0.749	0.034	0.776	0.042	0.720	*−* *0.262*	*0.028*
cg07349208	0.167	0.156	0.095	0.431	− 0.112	0.338	0.068	0.571

*PFC* prefrontal cortex, *EC* entorhinal cortex, *STG* superior temporal gyrus, *CER* cerebellum

Significant correlations in italics

## Discussion

To the best of our knowledge, our study is the first to investigate epigenome-wide methylation changes over time in BD patients. Moreover, it is the first to explore methylation changes related to non-traumatic stressful life events on an epigenome-wide scale. Although no locus withstood correction for multiple testing, our suggestive findings and secondary analyses provide limited evidence supporting a role of DNAm in association with non-traumatic life events in chronic BD patients.

We identified a single, suggestively significant, CpG site associated with total LEQ scores, mapping to *POU6F2*, which has been associated with several psychiatric traits as well as intelligence and educational attainment. More specifically, genome-wide association studies have identified *POU6F2* risk variants associated with psychological distress (Koshimizu et al. 2019), feeling emotionally hurt (Nagel et al. 2018), schizophrenia (Goes et al. 2015), autism (Anney et al. 2010), educational attainment (Lee et al. 2018; Okbay et al. 2016) and intelligence (Hill et al. 2018; Davies et al. 2018). Additionally, in a longitudinal investigation of DNAm changes preceding adolescent psychotic experiences, DNAm of the CpG site cg11604728 (*POU6F2*) measured at age 15–17 was among the top 20 CpG sites indicative of psychotic experiences at age 18 (Roberts et al. 2019). Furthermore, *POU6F2* is highly expressed in the brain with the highest expression found in the frontal cortex (Additional file 1: Fig. S13) and methylation of our suggestive CpG site in blood is correlated with methylation in brain tissue across multiple brain regions. Interestingly, another of our top 20 CpG sites (cg26822318) falls in proximity to the *FER1L6* gene, of which a variant (rs4870888) has been associated with suicide attempts in a meta-analysis of major depressive disorder, schizophrenia and BD (Mullins et al. 2019). Furthermore, another GWAS reported a *FER1L6* variant (rs10481151) suggestively associated with cognitive performance (Need et al. 2009).

At the current sample size, our study provides only minimal evidence supporting an association between methylation of individual CpGs and non-traumatic, recent stressful life events in BD. These findings, however, corroborate other reports of a limited role of DNAm with non-traumatic stress (Marzi et al. 2018). Noteworthy, a recent study reported hypermethylation of *KITLG* associated with childhood trauma in healthy controls (*n* = 91) but not in bipolar patients (*n* = 50) (He et al. 2018). Although the mechanistic role of DNAm in the phenotypic expression of early life adversities is well established in the literature, other mechanisms may be responsible in adulthood and in association with subsequent events. This notion aligns with theories such as Post’s kindling hypothesis and the decay model which suggest a higher impact of life events on first episode than on subsequent episodes in BD (Aldinger and Schulze 2017; Kemner et al. 2015; Hillegers et al. 2004). Furthermore, it must be considered whether positive epigenetic associations with life events could be disorder-specific, genotype-dependent, associated with specific trauma exposure, age groups, sex and/or tissues measured (Marzi et al. 2018; Vinkers et al. 2015; Uddin et al. 2010; Boks et al. 2015; Smith et al. 2011; Mehta et al. 2017). While there is no gold standard for life stress measurements, differences in how to quantify stress may also have a major effect on findings (Johnson 2005; Bender and Alloy 2011; Monroe 2008; Dohrenwend 2010; Brown and Harris 2012).

The main strength of our study is its longitudinal design, allowing for repeated measures within individuals and to investigate methylation changes over time and in relation to symptomatology and stressful life events. To the best of our knowledge, this is the first study to collect repeated epigenome-wide methylation measures in bipolar patients. Furthermore, our study paid attention to critical confounding factors which often lead to spurious findings. For example, the use of methylation-based smoking scores better controls for the extent of smoking throughout the lifetime than the use of self-reported smoking measures (Elliott et al. 2014; Shenker et al. 2013). Finally, in contrast to most other studies, we have included an exploratory epigenome-wide approach.

Despite the strengths of our study, several limitations need to be addressed. First, our study was limited by our small sample size which makes identifying subtle differences in methylation difficult. Taking power into consideration, and as an attempt to address the inherent multiple testing problem associated with EWAS, we limited our EWAS to only the most variable CpG sites according to MAD scores. While the fact that not a single site-specific association in DNAm survived correction for multiple testing could reflect the limited statistical power of our small sample, it may also be related to an overly conservative multiple testing correction considering the lack of variability in methylation at many CpGs and spatial correlation of methylation with nearby sites (Walker et al. 2016; Lunnon et al. 2015). A recent study estimated there are approximately 530,000 independent tests in a whole blood EPIC array DNAm study. Accordingly, they proposed a corrected significance threshold of 9.42 × 10^−8^ to be used as a standard threshold for future EWAS based on the EPIC array (Mansell et al. 2019). Furthermore, the study introduced a freely available online tool which allows users to perform power calculations to guide sample sizes, accounting for the individual properties of each DNAm site and using their empirically derived significance threshold. According to their tool, an effect size of just 1% difference between cases and controls would require a sample of 1000 participants, for only a third of methylation sites to have > 80% power. We observed an effect size below 5% in our study (based on median split) for our most significantly associated site, indicating that our study is nevertheless underpowered. Future studies should take advantage of this tool to assess, a priori, required sample sizes according to their expected effect sizes. Furthermore, complementary systems biology approaches such as weighted gene co-methylation network analysis (WGCNA) could be beneficial for studies with limited sample sizes, providing more insight into the functional role of altered DNAm (Langfelder and Horvath 2008).

Another limitation is in relation to the fact that our sample represents a cohort of chronic BD patients which likely influenced our investigation of epigenetic aging related to symptom ratings over time. The chronicity of patients may also confound our findings with regards to the heterogenous treatments patients have received over the years. To acknowledge this critical factor, we conducted a sensitivity analysis excluding those subjects not taking psychotropic drugs at the time of testing, however, this also did not lead to significant results. One must also consider the possible recall and desirability biases associated with self-rating questionnaires like the LEQ and CTS. Lastly, little is known about the temporal stability of epigenetic markers (Byun et al. 2012; Talens et al. 2010). We cannot be sure whether the time interval of 1 year was too long or short to observe dramatic methylation changes or at what time window following exposure to stressful life events one might observe changed methylation profiles.

## Conclusions

BD is a multifactorial psychiatric illness, and for many patients full interepisodic remission never occurs (Sam et al. 2019). Stressful life events have been associated with a worse course of BD (Aldinger and Schulze 2017) and there remains a need to better understand the mechanisms which allow these stressors to bring about phenotypic change. Our study provides limited evidence supporting an association between DNAm and recent, non-traumatic stressful life events in BD patients. As findings in clinical populations have been inconsistent, there is still much to be understood especially with regards to the temporal nature of environmentally induced DNA modifications. Future larger studies of well-characterized patients, longitudinal in design, are warranted.

## Supplementary information

**Additional file 1.** Supporting methods, Figures S1-S13, and Tables S1-S3.

## Data Availability

Participants of the PsyCourse study have consented to the sharing of their pseudonymized data with other researchers and research consortia. Thus, PsyCourse data will be made available to bona fide researchers collaborating with us given a mutually agreed written memorandum of understanding has been signed.

## Abbreviations

BD: bipolar disorder
GWAS: genome-wide association studies
DNAm: DNA methylation
CpG: cytosine-guanine dinucleotides
HPA: hypothalamic–pituitary–adrenal
LEQ: Life Events Questionnaire
CTS: Childhood Trauma Screener
PANSS: Positive and Negative Syndrome Scale
GAF: Global Assessment of Functioning
YMRS: Young Mania Rating Scale
IDS-C_30_: Inventory of Depressive Symptomatology
FunNorm: functional normalization
EWAS: epigenome-wide association analysis
BMIQ: beta-mixture quantile dilation
LMM: linear mixed-effects model
FDR: false discovery rate
MAD: median absolute deviation
AA: age acceleration
GO: gene ontology
QQ: quantile-quantile
WGCNA: weighted gene co-methylation network analysis
