# Supplementary material for: The role of environmental stress and DNA methylation in the longitudinal course of bipolar disorder
Source: Int J Bipolar Disord. 2020 Feb 12;8:9. doi: 10.1186/s40345-019-0176-6 (PMC7013010; doi:10.1186/s40345-019-0176-6)
Supplement: Supplementary file 1 — Additional file 1. Supporting methods, Figures S1-S13, and Tables S1-S3. [file 40345_2019_176_MOESM1_ESM.docx]

The role of environmental stress and DNA methylation in the longitudinal course of bipolar disorder

Ashley L. Comes^1,2,*^, Darina Czamara^3^, Kristina Adorjan^1,4^, Heike Anderson-Schmidt^1,5^, Till F. M. Andlauer^3,6^, Monika Budde^1^, Katrin Gade^5^, Maria Hake^1^, Janos L. Kalman^1,2,4^, Sergi Papiol^1,4^, Daniela Reich-Erkelenz^1^, Farah Klöhn-Saghatolislam^1,4^, Sabrina K. Schaupp^1^, Eva C. Schulte^1,4^, Fanny Senner^1,4^, Georg Juckel^7^, Max Schmauß^8^, Jörg Zimmermann^9^, Jens Reimer^10^, Eva Reininghaus^11^, Ion‐George Anghelescu^12^, Carsten Konrad^13^, Andreas Thiel^13^, Christian Figge^14^, Martin von Hagen^15^, Manfred Koller^16^, Detlef E. Dietrich^17-19^, Sebastian Stierl^20^, Harald Scherk^21^, Stephanie H. Witt^22^, Sugirthan Sivalingam^23^, Franziska Degenhardt^23^, Andreas J. Forstner^23-26^, Marcella Rietschel^22^, Markus M. Nöthen^23^, Jens Wiltfang^5,27,28^, Peter Falkai^4^, Thomas G. Schulze^1,4,a^ & Urs Heilbronner^1,a^

Author Affiliations:

^1^Institute of Psychiatric Phenomics and Genomics, University Hospital, LMU Munich, Munich, 80336, Germany; ^2^International Max Planck Research School for Translational Psychiatry (IMPRS-TP), Munich, 80804, Germany; ^3^Department of Translational Research in Psychiatry, Max Planck Institute of Psychiatry, Munich, 80804, Germany; ^4^Department of Psychiatry and Psychotherapy, University Hospital, LMU Munich, Munich, 80336, Germany; ^5^Department of Psychiatry and Psychotherapy, University Medical Center Göttingen, Göttingen, 37075, Germany; ^6^Department of Neurology, Klinikum rechts der Isar, School of Medicine, Technical University of Munich, Munich, 81675, Germany; ^7^Department of Psychiatry, Ruhr University Bochum, LWL University Hospital, Bochum, 44791, Germany; ^8^Department of Psychiatry and Psychotherapy, Bezirkskrankenhaus Augsburg, University of Augsburg, Augsburg, 86156, Germany; ^9^Psychiatrieverbund Oldenburger Land gGmbH, Karl-Jaspers-Klinik, Bad Zwischenahn, 26160, Germany; ^10^Department of Psychiatry and Psychotherapy, University Medical Center Hamburg-Eppendorf, Hamburg, D-20246, Germany; ^11^Department of Psychiatry and Psychotherapeutic Medicine, Research Unit for Bipolar Affective Disorder, Medical University of Graz, Graz, 8036, Austria; ^12^Department of Psychiatry, Dr. Frontheim-Mental Health, Liebenburg, 38704, Germany; ^13^Department of Psychiatry and Psychotherapy, Agaplesion Diakonieklinikum, Rotenburg, 27356, Germany; ^14^Karl-Jaspers Clinic, European Medical School Oldenburg-Groningen, Oldenburg, 26160, Germany; ^15^Clinic for Psychiatry and Psychotherapy, Clinical Center Werra-Meißner, Eschwege, 37269, Germany; ^16^Asklepios Specialized Hospital, Göttingen, 37081, Germany; ^17^AMEOS Clinical Center Hildesheim, Hildesheim, 31135, Germany; ^18^Center für Systems Neuroscience (ZSN) Hannover, Hannover, 30559, Germany; ^19^Department of Psychiatry, Medical School of Hannover, Hannover, 30625, Germany; ^20^Psychiatric Hospital Lüneburg, Lüneburg, 21339, Germany; ^21^AMEOS Clinical Center Osnabrück, Osnabrück, 49088, Germany; ^22^Department of Genetic Epidemiology in Psychiatry, Central Institute of Mental Health, Medical Faculty Mannheim, University of Heidelberg, Mannheim, 68159, Germany; ^23^Institute of Human Genetics, University of Bonn, School of Medicine & University Hospital Bonn, Bonn, 53127, Germany; ^24^Center for Human Genetics, University of Marburg, Marburg, 35033, Germany; ^25^Department of Biomedicine, University of Basel, Basel, 4031, Switzerland; ^26^Department of Psychiatry (UPK), University of Basel, Basel, 4002, Switzerland; ^27^German Center for Neurodegenerative Diseases (DZNE), Göttingen, 37075, Germany; ^28^iBiMED, Medical Sciences Department, University of Aveiro, Aveiro, 3810-193, Portugal

^a^ Authors contributed equally

^*^Corresponding Author: Ashley L. Comes, Institute of Psychiatric Phenomics and Genomics, University Hospital, LMU Munich; Nussbaumstrasse 7; 80336 Munich, Germany; email, [Ashley.Comes@med.uni-muenchen.de](mailto:Ashley.Comes@med.uni-muenchen.de); tel., +49 089 4400 55556

Supplementary methods

*Batch effects.* We used R version 3.4.4 to randomly draw samples from selected individuals stratified by groups (age, sex, and exposures of interest) and to assign them to batches (e.g. different microarray chips). Standard statistical tests were then used to assess if the putative technical batches (e.g. plates, microarray chips, chip rows/columns) were significantly associated with age, gender or exposure of interest. This procedure was repeated for up to 10000 permutations and the combination of samples that produced the least significant associations was selected. As within-individual variability over time was explored, baseline and visit 3 samples for each individual were loaded onto the same chip. Post hoc tests were then run on the selected combination of samples to confirm no significant associations with other variables, namely diagnosis (Bipolar I/II) or disease severity.

*Blood-brain methylation correlation.* The freely available Blood Brain DNA Methylation Comparison Tool (<http://epigenetics.iop.kcl.ac.uk/bloodbrain/>) (1) was used to determine the likely correlation between DNAm in blood with four different brain regions (i.e., prefrontal cortex, entorhinal cortex, superior temporal gyrus and cerebellum) for the most suggestive CpGs associated with total LEQ scores.

**Table S1.** Reported childhood trauma type

| Childhood trauma type |  | n (%) |
| --- | --- | --- |
| Abuse only |  | 22 (45.8) |
| Neglect only |  | 6 (12.5) |
| Number of traumas |  |  |
| 1 |  | 22 (45.8) |
| 2 |  | 11 (22.9) |
| 3 |  | 8 (16.7) |
| 4 |  | 1 (2.1) |
| 5 |  | 1 (2.1) |
| Missing complete CTS |  | 5 (10.4) |
| CTS single items | Trauma threshold |  |
| “. . . I had the feeling to be loved” | “Seldom” or “Not at all” | 19 (39.6) |
| “. . . persons in my family hit me so hard that I bruised” | “Sometimes” to “Very often” | 16 (33.3) |
| “. . . I had the feeling someone in my family hated me” | “Sometimes” to “Very often” | 24 (59.0) |
| “. . . someone harassed me sexually” | “Seldom” to “Very often” | 15 (31.2) |
| “. . . there was someone who took me to the doctor when I needed it” | “Seldom” or “Not at all” | 10 (20.8) |

**Table S2.** Probe filtering

| **CpGs probes EPIC** | **866,238** |
| --- | --- |
| Low detection p-values | 6529 |
| Bead count < 3 | 17315 |
| X and Y probes | 19096 |
| CpGs with SNPs | 29310 |
| Cross-reactive probes | 37968 |
| Non-specific probes | 2769 |
| **CpGs after filtering** | **753,251** |


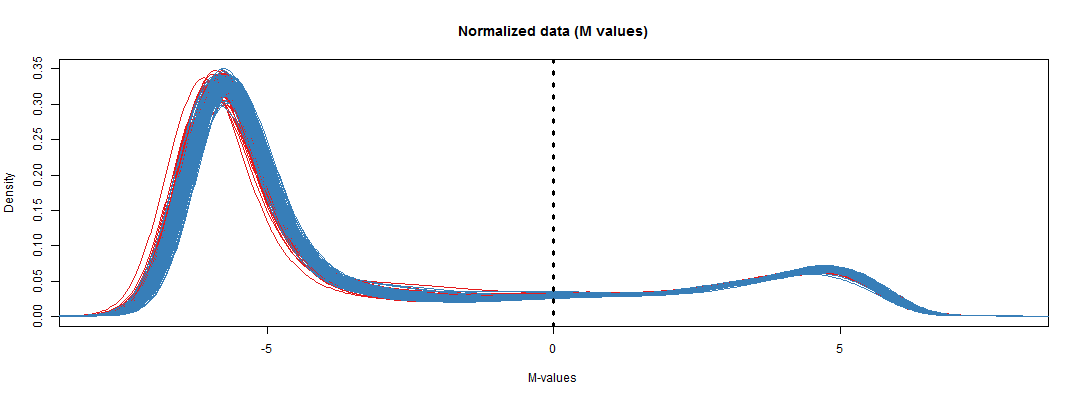


**Figure S1*.*** *M*-Value densities before and after functional normalization.

**Figure S2.** Inspection of batch effects through principle component analysis plots before and after batch correction with ComBat


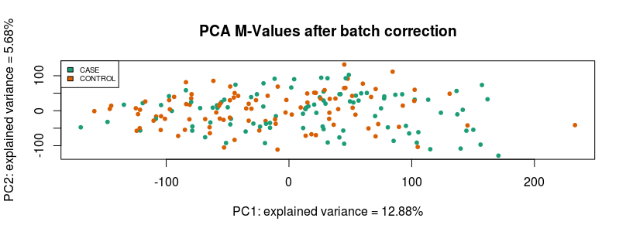


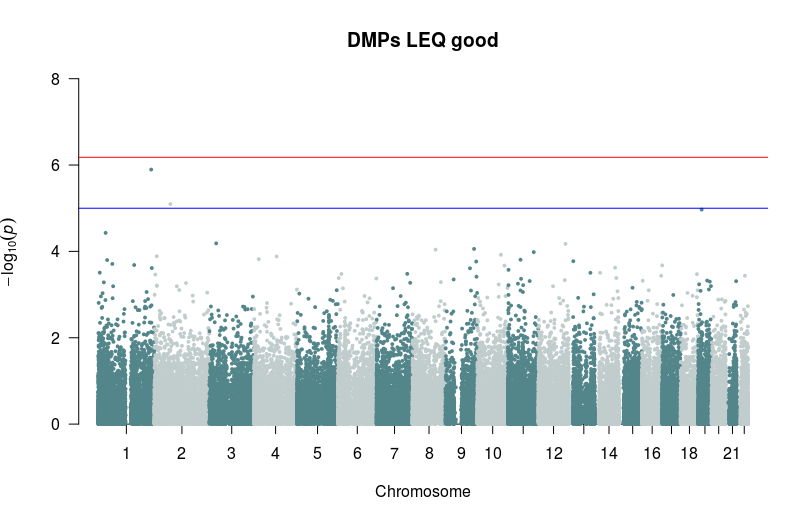


**Figure S3.** **Manhattan plot.** The Manhattan plot depicts the association between DNA methylation and LEQ “good” scores (n = 191). The horizontal red line represents the epigenome-wide significant threshold for this study (p < 6.6×10^-7^) and the blue line represents a suggestive significance threshold (p < 1.0×10^-5^).


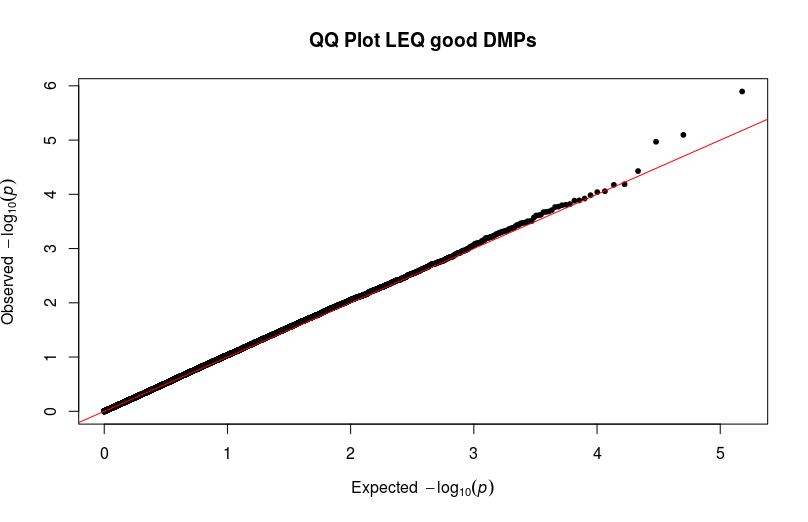


**Figure S4. QQ plot.** The QQ plot shows no evidence for inflation or bias in the association analysis of DNA methylation with “good” LEQ scores (Lambda = 1.04).


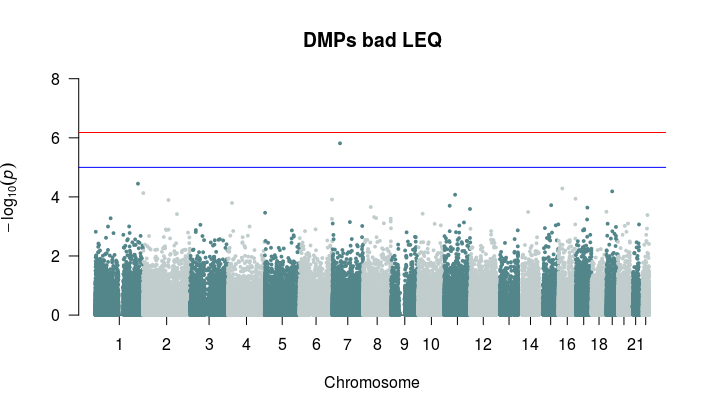


**Figure S5. Manhattan plot.** The Manhattan plot depicts the association between DNA methylation and “bad” LEQ scores (n = 191). The horizontal red line represents the epigenome-wide significant threshold for this study (p < 6.6×10^-7^) and the blue line represents a suggestive significance threshold (p < 1.0×10^-5^).


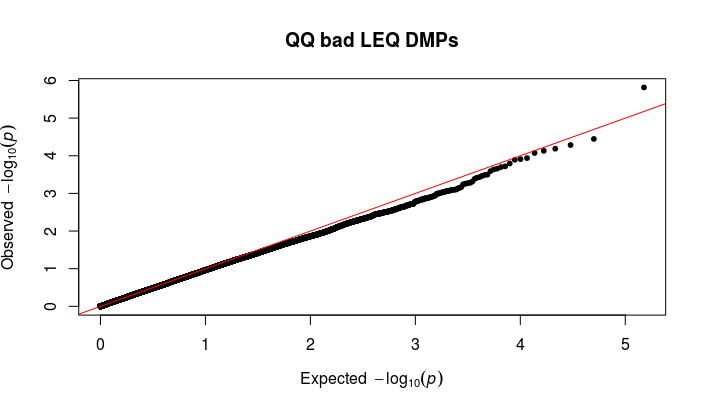


**Figure S6.** **QQ plot.** The QQ plot shows no evidence for inflation or bias in the association analysis of DNA methylation with “bad” LEQ scores (Lambda = 0.96).


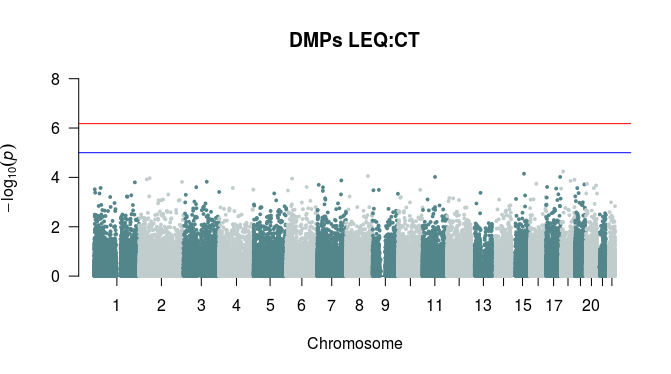


**Figure S7. Manhattan plot.** The Manhattan plot depicts the association between DNA methylation and the interaction between childhood trauma and total LEQ scores (n = 191). The horizontal red line represents the epigenome-wide significant threshold for this study (p < 6.6×10^-7^) and the blue line represents the suggestive significance threshold (p< 1.0×10^-5^).


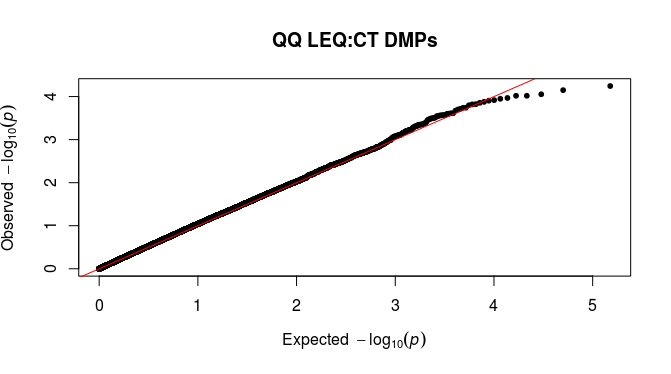


**Figure S8. QQ plot.** The QQ plot shows no evidence for inflation or bias in the association analysis of DNA methylation with the interaction between childhood trauma and total LEQ scores (Lambda = 1.10).


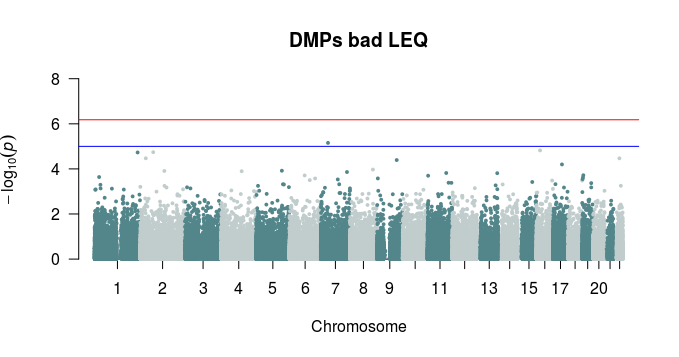


**Figure S9.** **Manhattan plot.** The Manhattan plot depicts a sensitivity analysis testing the association between DNA methylation and total LEQ scores after removing patients not taking psychotropic drugs at the time of testing (n = 186). The horizontal red line represents the epigenome-wide significant threshold for this study (p < 6.6×10^-7^) and the blue line represents the suggestive significance threshold (p < 1.0×10^-5^).


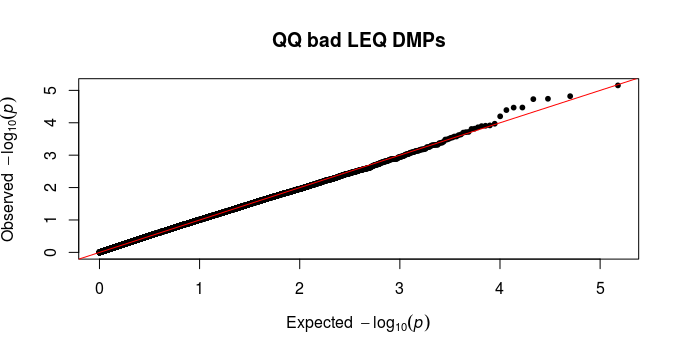


**Figure S10. QQ plot.** The QQ plot shows no evidence for inflation or bias in the association analysis of DNA methylation with total LEQ scores after removal of patients not on taking psychotropic drugs at the time of testing (Lambda = 1.06).


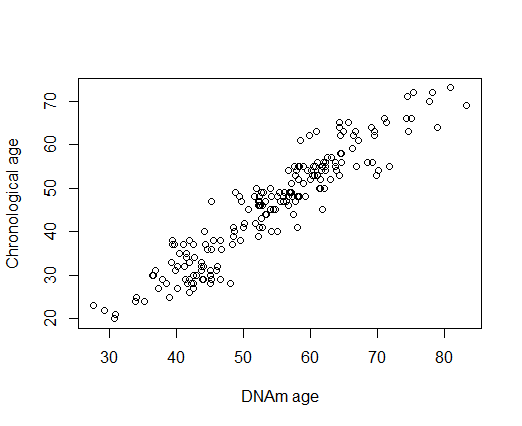


**Figure S11.** **Scatterplot.** The scatterplot illustrates the significant and positive correlation between DNA methylation age (DNAm age in years, calculated based on the Horvath algorithm) and chronological age (years) at baseline (Spearman’s ρ = 0.94).

**Table S3.** GOmeth enrichment analysis

| Biological process |  | N | DE | P.DE | FDR |
| --- | --- | --- | --- | --- | --- |
| homophilic cell adhesion via plasma membrane adhesion molecules |  | 136 | 54 | 7.28E-05 | 1 |
| spindle midzone assembly |  | 8 | 6 | 0.001 | 1 |
| cell-cell adhesion via plasma-membrane adhesion molecules |  | 199 | 64 | 0.001 | 1 |
| leukotriene biosynthetic process |  | 10 | 6 | 0.002 | 1 |
| exploration behavior |  | 22 | 11 | 0.003 | 1 |
| maintenance of unfolded protein |  | 3 | 3 | 0.003 | 1 |
| maintenance of unfolded protein involved in ERAD pathway |  | 3 | 3 | 0.003 | 1 |
| regulation of epithelial cell migration |  | 154 | 41 | 0.004 | 1 |
| myelination |  | 87 | 26 | 0.004 | 1 |
| vesicle targeting, trans-Golgi to endosome |  | 2 | 2 | 0.004 | 1 |
| regulation of endothelial cell migration |  | 110 | 30 | 0.005 | 1 |
| negative regulation of osteoclast development |  | 5 | 4 | 0.006 | 1 |
| ensheathment of neurons |  | 90 | 26 | 0.006 | 1 |
| axon ensheathment |  | 90 | 26 | 0.006 | 1 |
| positive regulation by host of viral transcription |  | 9 | 5 | 0.007 | 1 |
| cellular response to arsenic-containing substance |  | 12 | 5 | 0.007 | 1 |
| germinal center B cell differentiation |  | 2 | 2 | 0.007 | 1 |
| hormone-mediated apoptotic signaling pathway |  | 3 | 3 | 0.007 | 1 |
| positive regulation of axon extension |  | 26 | 11 | 0.007 | 1 |
| nervous system development |  | 1747 | 369 | 0.008 | 1 |
| Abbreviations: N – number of genes in the GO; DE – number of genes that are differentially methylated; P.DE – *p*-value for the over-representation of the GO term | | | | | |
|  |  |  |  |  |  |


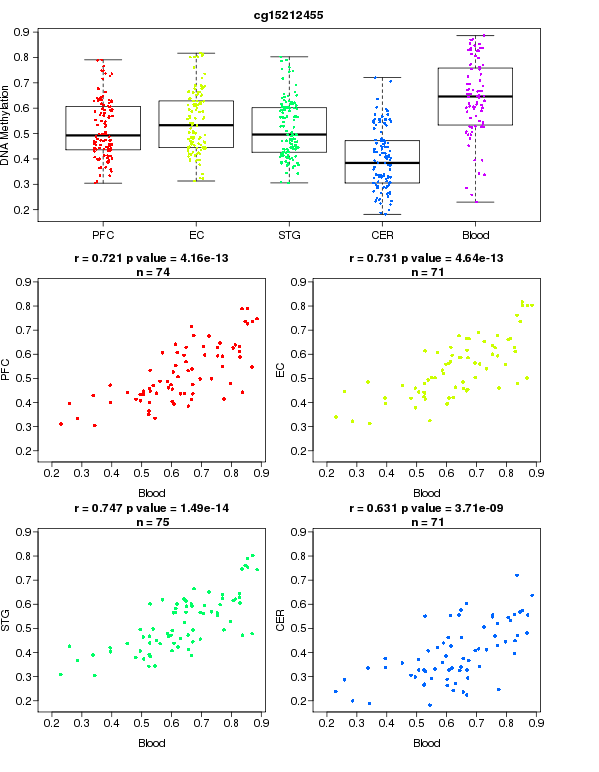


**Figure S12. Blood-brain methylation correlation.** Publicly available data from Hannon et al. was used to assess cg15212455 DNA methylation patterns across blood, the prefrontal cortex (PFC), entorhinal cortex (EC), superior temporal gyrus (STG), and cerebellum (CER).

*
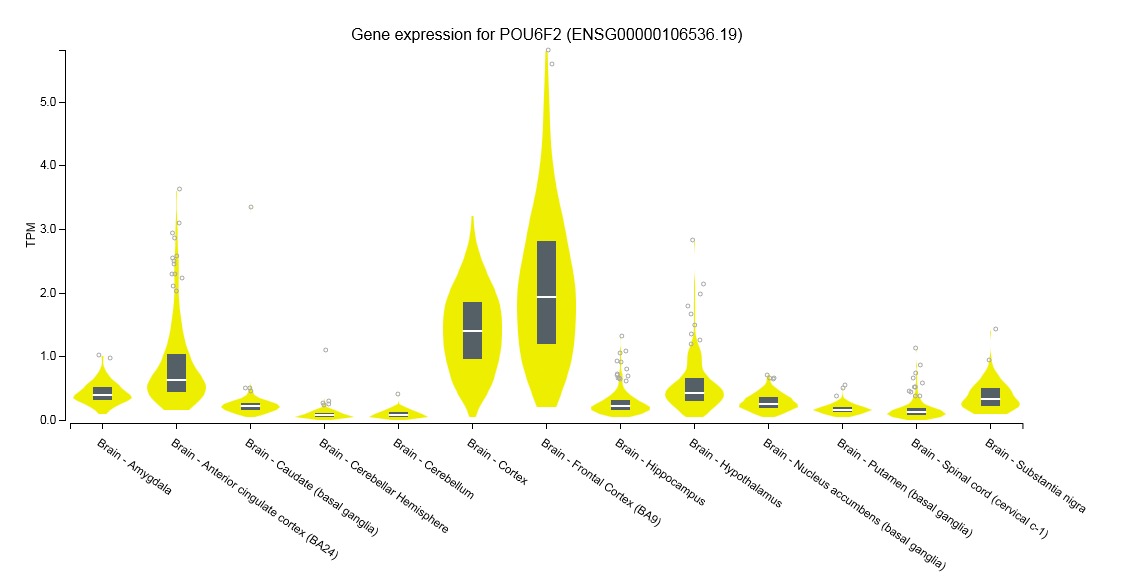
*

**Figure S13** **Gene expression patterns of POU6F2 across multiple brain regions.** The freely available Genotype-Tissue Expression (GTEx) Project Database portal (www.gtexportal.org) was used to determine the expression patterns of POU6F2 across multiple tissues. The figure shows expression levels across multiple brain regions, showing the highest expression of POU6F2 in the frontal cortex.

**References**

1. Hannon E, Lunnon K, Schalkwyk L, Mill J. Interindividual methylomic variation across blood, cortex, and cerebellum: implications for epigenetic studies of neurological and neuropsychiatric phenotypes. Epigenetics. 2015;10(11):1024-32.
